# Non-Invasive Regional Neurochemical Profiling of Zebrafish Brain Using Localized Magnetic Resonance Spectroscopy at 28.2 T

**DOI:** 10.3390/molecules30214320

**Published:** 2025-11-06

**Authors:** Rico Singer, Wanbin Hu, Li Liu, Huub J. M. de Groot, Herman P. Spaink, A. Alia

**Affiliations:** 1 Leiden Institute of Chemistry, Leiden University, Einsteinweg 55, 2301 RA Leiden, The Netherlands; r.singer@lic.leidenuniv.nl (R.S.); groot_h@lic.leidenuniv.nl (H.J.M.d.G.); 2 Institute of Biology, Leiden University, Einsteinweg 55, 2301 RA Leiden, The Netherlands; w.hu@biology.leidenuniv.nl (W.H.); l.liu@biology.leidenuniv.nl (L.L.); h.p.spaink@biology.leidenuniv.nl (H.P.S.); 3 Institute of Medical Physics and Biophysics, Leipzig University, Härtelstr. 16-18, D-04107 Leipzig, Germany

**Keywords:** magnetic resonance spectroscopy, point-resolved spectroscopy, zebrafish, cerebral metabolites, 28.2 T ultra-high magnetic field strength

## Abstract

Localized ^1^H magnetic resonance spectroscopy (MRS) is a powerful tool in pre-clinical and clinical neurological research, offering non-invasive insight into neurochemical composition in localized brain regions. Zebrafish (*Danio rerio*) are increasingly being utilized as models in neurological disorder research, providing valuable insights into disease mechanisms. However, the small size of the zebrafish brain and limited MRS sensitivity at low magnetic fields hinder comprehensive neurochemical analysis of localized brain regions. Here, we investigate the potential of ultra-high-field (UHF) MR systems, particularly 28.2 T, for this purpose. This present study pioneers the application of localized ^1^H spectroscopy in zebrafish brain at 28.2 T. Point resolved spectroscopy (PRESS) sequence parameters were optimized to reduce the impact of chemical shift displacement error and to enable molecular level information from distinct brain regions. Optimized parameters included gradient strength, excitation frequency, echo time, and voxel volume specifically targeting the 0–4.5 ppm chemical shift regions. Exceptionally high-resolution cerebral metabolite spectra were successfully acquired from localized regions of the zebrafish brain in voxels as small as 125 nL, allowing for the identification and quantification of major brain metabolites with remarkable spectral clarity, including lactate, myo-inositol, creatine, alanine, glutamate, glutamine, choline (phosphocholine + glycerol-phospho-choline), taurine, aspartate, N-acetylaspartyl-glutamate (NAAG), N-acetylaspartate (NAA), and γ-aminobutyric acid (GABA). The unprecedented spatial resolution achieved in a small model organism enabled detailed comparisons of the neurochemical composition across distinct zebrafish brain regions, including the forebrain, midbrain, and hindbrain. This level of precision opens exciting new opportunities to investigate how specific diseases in zebrafish models influence the neurochemical composition of specific brain areas.

## 1. Introduction

Localized magnetic resonance spectroscopy (MRS) is a well-established, non-invasive technique for monitoring metabolic changes in human clinical studies and pre-clinical studies using animal models [1]. Unlike various other metabolic methodologies, localized MRS bypasses the need for laborious extraction procedures. Moreover, it facilitates precise selection of tissue regions, enabling in vivo monitoring of metabolites within their native environments. While localized MRS is utilized for various tissue types [2,3,4,5], clinical applications predominantly focus on brain tissue across a wide range of neurological disorders [6,7,8,9,10,11,12]. In this respect, brain metabolites show a remarkable sensitivity to deviations from physiological homeostasis induced by pathological conditions. Specific metabolites offer insights into distinct cells and their corresponding cellular activities. For instance, localized MRS enables quantification of glutamate (Glu) and γ-aminobutyric acid (GABA), the principal excitatory and inhibitory neurotransmitters in the brain, respectively [13,14]. Additionally, N-acetylaspartate (NAA), predominantly localized in neuronal cell bodies and axons, serves as a potential marker for monitoring neuronal integrity [15] and *myo*-inositol (m-Ins), chiefly situated in glial cells, functions as a potential marker for astrogliosis and neuroinflammation [16].

Zebrafish (*Danio rerio*) is an important model organism for several human diseases, including neurological disorders. For scientific inquiry, zebrafish offer distinct advantages, including cost-effectiveness and easy maintenance, coupled with a rapid developmental pace. Zebrafish embryos are particularly beneficial in developmental and toxicity studies at a cellular and molecular level [17,18,19,20,21,22]. The entire zebrafish genome has been sequenced [23], revealing 84% of genes linked to human diseases possess a corresponding homologous gene in the zebrafish genome. These similarities with humans allow for genetic manipulation to create transgenic zebrafish models to study human diseases. For neurological disorders, an extensive number of zebrafish models are available, including transgenic models of Alzheimer’s disease, Parkinson’s disease, brain tumors, and epilepsy [24,25,26,27,28]. Despite their considerable use in research, studies utilizing localized MRS involving zebrafish are rare. Previously, we presented the first study of localized MRS for in vivo analysis of cerebral metabolites in zebrafish at 9.4 T (400 MHz) [29]. High-resolution spectra were obtained, allowing for the identification and quantification of the most predominant metabolites, including creatine (Cr), Glu, NAA, m-Ins, glutamine (Gln), taurine (Tau), and GABA. However, the limited sensitivity of localized MRS, in combination with the small size of the zebrafish brain (few mm^3^), prevented the application from analyzing localized brain regions.

Recent developments in MR systems operating at high fields and ultra-high fields (UHF), offer remarkable potential in elucidating intricate details within the zebrafish brain using MRI [30]. Elevated signal-to-noise ratio (SNR) in combination with extremely high gradient strengths, unveiled previously unseen details in the zebrafish brain through non-invasive magnetic resonance imaging (MRI), including the identification of tiny white matter structures through diffusion-based MRI fiber tracking [30,31]. However, despite these recent breakthroughs, the application of localized MRS in the zebrafish brain at UHF remains untapped. Due to anticipated enhancements in SNR and improved baseline separation of metabolites, localized MRS at UHFs holds tremendous promise. However, it may also introduce specific challenges, notably increased chemical shift displacement (*CSD*) effects. *CSD*, also known as chemical shift misregistration or chemical shift artifacts, were first elucidated in 1984 though the registration shift in lipid signals [32]. These effects arise from the interplay between the offset Δ*v*_L_, i.e., the difference between the applied excitation frequency *v*_1_ and the specific Larmor frequencies of specific compounds *v*_x_, and the applied magnetic field gradients (*G*). *CSD* escalate with the magnetic field strength B_0_ due to the increase in Δ*v*_L_ and recede with stronger *G* due to the frequency-encoding bandwidth per pixel becoming larger [33]. Despite UHF MRI systems utilizing *G*_max_ far exceeding the typical strengths used in medical imaging systems [34,35], these gradients remain insufficient to fully compensate for the increase in Δ*v*_L_. Additionally, UHF systems are often used on small samples and at cellular resolution (~50 µm), making the chemical shift effect become relatively more pronounced. Consequently, chemical shift displacements will be enhanced in localized MRS utilizing UHF systems. Addressing this challenge requires sophisticated correction methods to accurately quantify metabolite concentrations and interpret spectroscopic data. In the current study, we utilized point-resolved spectroscopy (PRESS) [36]. The PRESS sequence, consisting of three RF pulses (90°-180°-180°), provides good SNR and relatively short scan times, although it is susceptible to CSD and B_1_ inhomogeneity making it less optimal at ultra-high fields where these effects are amplified. In this regard, alternative sequences such as stimulated echo acquisition mode (STEAM) and image-selected in vivo spectroscopy (ISIS) are available. However, each of these comes with their own limitations. In the case of STEAM, while its utilization of three 90֯ slice-selective pulses make it less susceptible to CSD and B_1_ inhomogeneity, it only provides half the SNR compared to PRESS [37], making it less effective for small ROIs. For ISIS, eight separate acquisitions are required, thus providing significantly less SNR in the same measurement time as PRESS [38]. Alternatively, localization by adiabatic selective refocusing (LASER) can be employed; this technique uses adiabatic pulses, making it robust for B1 inhomogeneity and reducing CSD [39]. However, LASER requires advanced hardware/software, which are not widely available on standard systems. In contrast, PRESS is easier to set up and optimize because it uses conventional 90°–180° slice-selective pulses, thus less technically demanding and reduces set up time. It is a well-established sequence, making it the method of choice in the current work.

This study presents the first successful demonstration of localized MRS at 28.2 T, currently the highest commercially available magnetic field strength. Beyond parameterizing key sequence parameters (including the excitation frequency (*v*_1_) and transmitter RF pulse bandwidth (*tBW*)) to mitigate chemical shift displacement artifacts, we systematically assessed the influence of echo time and voxel size on spectral integrity and metabolite detectability. Utilizing optimized parameters, our approach enabled localized MRS in remarkably small brain regions, with total acquisition times ranging from just 15 to 30 min, bringing it in the range required for in vivo applications. Our protocol allowed for the quantification of major brain metabolites and the acquisition of high-quality spectra from voxel volumes as small as 125 nL. Our localized MRS at 28.2 T provides unprecedented spatial resolution, enabling clear identification and quantification of multiple metabolites with remarkable spectral clarity in tiny brain regions, thus paving the way for future studies involving comprehensive investigations of neurochemical profiles in zebrafish brain, utilizing various zebrafish models of human diseases.

## 2. Results and Discussion

In this study, we present the first localized MRS results of the zebrafish brain at the state-of-the-art magnetic field strength of 28.2 T. While non-invasive MRI and MRS holds great promise for exploring brain pathology in zebrafish models, achieving the high resolution and signal-to-noise ratio (*SNR*) necessary to study metabolites in tiny brain regions remains a significant challenge. In this work, we optimized and successfully implemented localized MRS techniques at 28.2 T to gain insight into various brain regions in young adult zebrafish.

### 2.1. Mitigating the Chemical Shift Displacement at UHF

*CSD* effects become more pronounced at elevated magnetic field strengths [40].

**Figure 1:**
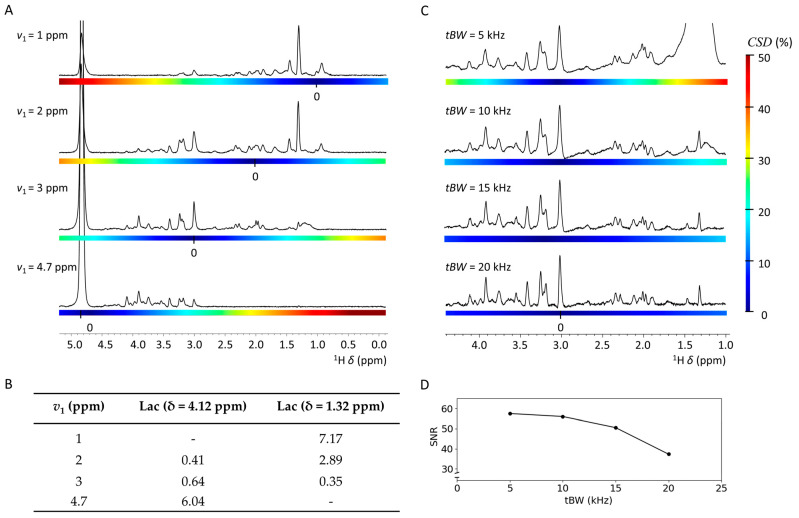
Impact of the excitation frequency *v*_1_ and transmitter RF pulse bandwidth *tBW* on acquired single voxel localized ^1^H MRS spectra from adult zebrafish mid-brain at 28.2 T. (**A**) PRESS spectra acquired at various *v*_1_ and its impact on magnitude of chemical shift displacement, *CSD*, indicated by color bar. (**B**) Estimated lactate concentration (relative to the tCr) as a function of the excitation frequency *v*_1_. Insufficient signal for estimation is indicated by (-). (**C**) PRESS spectra at various *tBW* and its impact on magnitude of chemical shift displacement, *CSD* indicated by color bar. (**D**) *SNR* of the tCr signal at *δ* = 3.03 ppm as a function of *tBW*. To facilitate direct comparison, spectral intensities were normalized relative to the tCr signal at *δ* = 3.03 ppm. Acquisition details, unless stated otherwise: *TR* = 2000 ms, *TE* = 15 ms, *ns* = 1024, voxel size 0.8 × 0.8 × 0.8 mm^3^ with *V*_voxel_ = 512 nL, 2048 acquisition points, acquisition bandwidth 11,904 Hz/9.91 ppm, 4 dummy scans, *v*_1_ = 3 ppm, *tBW* = 10 kHz. Water suppression and outer volume suppression were applied as specified in the Materials and Methods Section.

To mitigate the impact of chemical shift displacement, excitation frequency *v*_1_ can be adjusted to be in closer proximity to the chemical shift of the metabolites of interest. In Figure 1A, we explored the impact of *v*_1_ on the extent of *CSD* at 28.2 T. An adjustment of *v*_1_ by ±1 ppm, equivalent to ±1200 Hz at 28.2 T, led to notable changes in the obtained spectra. A near-total loss of metabolite signals occurred in spectral regions where *CSD* surpassed 30%, as demonstrated at excitation frequencies *v*_1_ = 1 ppm or *v*_1_ = 4.7 ppm. Moreover, *CSD* caused noticeable changes in metabolite signal intensities with chemical groups at different chemical shifts *δ*. For metabolites exhibiting pronounced internal chemical shift differences between their functional groups, including lactate, the displacement effect critically impacted quantification. This effect is clearly demonstrated in Figure 1B. A concentration of 0.41 relative to tCr was observed for the lactate methine signal at *δ* = 4.1 ppm, while a concentration of 2.89 relative to tCr was observed for the lactate methyl signal at *δ* = 1.3 ppm, utilizing *v*_1_ = 2 ppm. Therefore, to ensure accurate metabolite quantification during comparative analyses, it is essential to consistently select the same chemical group. For brain analysis, metabolites of interest are found in the chemical shift range between the lactate methine signal at δ = 4.1 ppm and the lactate methyl signal at δ = 1.3 ppm [41]. These include Glu, Gln, m-Ins, Tau, Cr, GABA, and NAA. Hence, unless stated otherwise, localized MRS acquisitions in this work were performed with *v*_1_ = 3 ppm to balance *CSD* across the spectrum.

Besides *v*_1_, the transmitter RF pulse bandwidth *tBW* of the excitation and refocusing pulses impact the magnitude of *CSD*. In Figure 1C, we probed the impact of *tBW* on the localized MRS spectra, acquired with *v*_1_ = 3 ppm. At *tBW* = 5 kHz, significant lipid signals were observed around *δ* = 1.3 ppm. The zebrafish brain is encapsulated by lipids, especially fat. To analyze the fat distribution around the zebrafish brain, we performed fat mapping using chemical shift selective imaging. As shown in Supplementary Figure S1, a significant amount of fat was present around the zebrafish brain. In our localized spectra shown in Figure 1C, a huge signal from fat was observed at around δ = 1.3 ppm. This fat signal overlaps with the other metabolite signals, such as the lactate methyl signal (δ = 1.32 ppm) and alanine methyl signal (*δ* = 1.47 ppm). Although the PRESS voxel was fully placed inside the brain, *CSD* caused the detection of lipid signals originating from outside the brain.

For *tBW* = 10 kHz, the interfering lipid signal was still observed, although significantly reduced compared to *tBW* = 5 kHz. For *tBW* = 15 kHz or 20 kHz, the lipid signals were no longer observed, allowing for the identification and quantification of the lactate and alanine (Ala) signals at *δ* = 1.32 ppm and *δ* = 1.47 ppm, respectively. Utilizing a tBW of 20 kHz ensures nearly all metabolites remain within a CSD of 10%, as recommended [41], with the only exception being the lactate signal at 1.3 ppm, which reaches a CSD of 10.2%. However, we observed that increasing *tBW* leads to diminished SNR. As illustrated in Figure 1D, a significant decrease in *SNR* of approximately 25% was observed increasing the *tBW* = 15 kHz to 20 kHz, possibly due to exceeding of the maximum peak B_1_ of the RF coil, resulting in inaccuracies in the applied PRESS flip angles. Consequently, for further measurements *tBW* = 15 kHz was selected, provided a significant reduction in *CSD* while maintaining sufficient *SNR* and with a *CSD* below 10% for most metabolites of interest.

### 2.2. Localized MRS at Variable Echo Times

**Figure 2:**
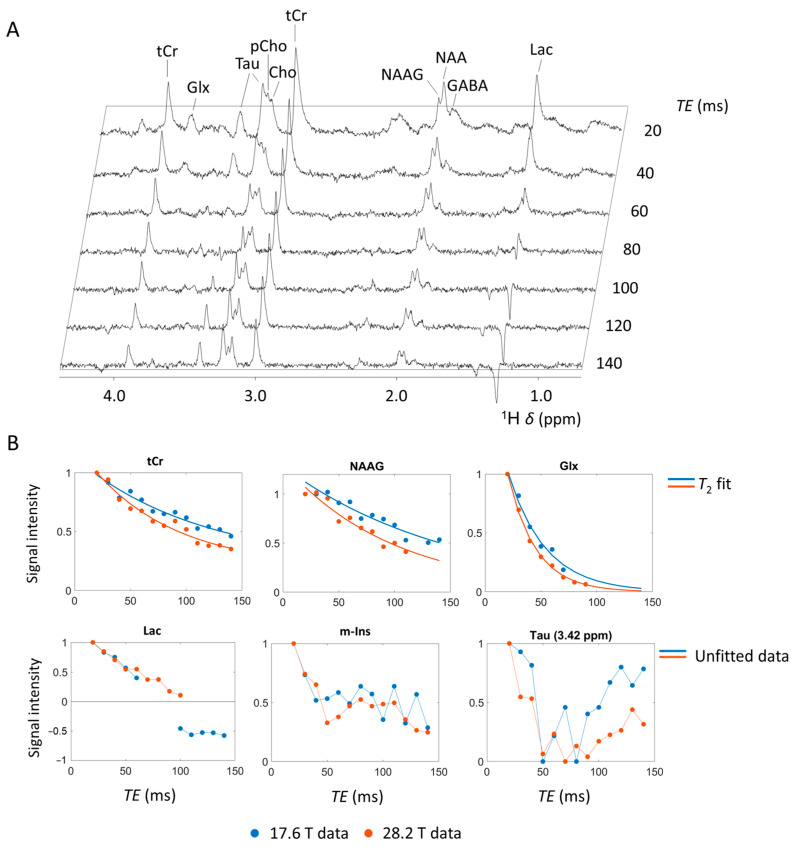
Impact of the echo time on the acquired single voxel localized ^1^H MRS spectra from adult zebrafish mid-brain at 17.6 T and 28.2 T. (**A**) Single voxel localized MRS spectra acquired for echo times ranging from 20 to 140 ms at 17.6 T. (**B**) (**upper**) Signal decay observed for cerebral metabolites with an apparent consistent mono-exponential decrease with echo time, including tCr, NAAG, and Glx (Gln + Glu). Mono-exponential decay curve from *T*_2_ relaxation time estimation fitted through data. (**B**) (**lower**) Signal decay of metabolites with a more complex signal intensity evolution contributed to J-coupling induced signal dephasing, including Lac, m-Ins, and Tau. Acquisition details: *TR* = 2000 ms, *TE* = [15, 20, 30, 40, 50, 60, 70, 80, 90, 100, 110, 120, 130, or 140] ms, *ns* = 1024, a voxel size of 0.8 × 0.8 × 0.8 mm^3^ with *V*_voxel_ = 512 nL, 2048 acquisition points, acquisition bandwidth 11,904 Hz/9.91 ppm, *tBW* = 10 kHz for all RF pulses, 4 dummy scans, *v*_1_ = 3 ppm. Water suppression and outer volume suppression were applied as specified in the Materials and Methods Section.

Echo time plays a pivotal role in the acquired metabolic profile and warrants careful consideration during experimental design. A well-established effect is the diminishing of signal intensities at elevated echo times as a consequence of transverse relaxation processes (*T*_2_). Hence, shorter echo times are frequently favored to enhance *SNR*. Nonetheless, the deliberate selection of longer echo times can yield substantial enhancements in metabolite identification and quantification. Furthermore, applying a range of echo times allows for the estimation of *T*_2_ relaxation times of individual metabolites, providing insight into changes within their respective environments. For instance, in Alzheimer’s disease patients and models, a significant increase in the *T*_2_ of NAA was reported, while no significant change was observed in the *T*_2_ of water in the same tissue [42,43]. Unlike the *T*_2_ of water, which reflects properties of both intracellular and extracellular spaces, NAA is confined solely to the intracellular space, thus furnishing more nuanced and compartment-specific details [42]. Figure 2A shows the cerebral metabolite profile at increasing echo times obtained at 17.6 T. A full comparison of the cerebral metabolite profile at both 17.6 T and 28.2 T is shown in Supplementary Figure S2. Beyond echo times of 60 ms, the lipid signals around *δ* = 0.9 ppm and *δ* = 2.0 ppm became indiscernible amidst the background noise, due to the relatively short *T*_2_ relaxation time of these macromolecules [44]. This facilitates improved identification and quantification of cerebral metabolites with chemical shifts overlapping with lipid signals, e.g., Lac, Ala, GABA, NAA, and N-acetyl aspartyl-glutamate (NAAG). In addition, water exhibits a shorter *T*_2_ relaxation time compared to most presented cerebral metabolites [45]. Consequently, the suppression of the broad and predominant water signal was notably improved at higher echo times. The signal intensity of various metabolite signals, including the tCr signal at *δ* = 3.91 ppm (^2^CH_2_) and *δ* = 3.03 ppm (N(CH_3_)), the NAA signal at *δ* = 2.01 ppm (^2^CH_3_), the NAAG signal at *δ* = 2.04 ppm (^2^CH_3_), the GABA signal at *δ* = 1.89 ppm (^3^CH_2_), and the Glx (Glu + Gln) signal at *δ* = 3.75 ppm (Glu ^2^CH and Gln ^2^CH) appeared to decrease exponentially with increasing echo time (see Figure 2B) which allowed for direct *T*_2_ estimation through mono-exponential line fitting, as we demonstrate in Supplementary Figure S2. At 28.2 T, several metabolites exhibited shorter *T*_2_ relaxation times compared to 17.6 T, as evidenced by a more rapid signal decay. The decline in *T*_2_ relaxation times with increased magnetic field strengths appears to contradict the Bloembergen–Purcell–Pound (BPP) dipolar relaxation theory [46]. However, our findings are in line with previous observations, showing the decline of *T*_2_ of (cerebral) metabolites at elevated magnetic field strengths in both humans [47,48,49] and animal models [43,50]. This apparent contradiction between theory and practical observations was previously attributed to diffusion-induced dephasing in microscopic susceptibility gradients, which becomes more pronounced at higher magnetic field strengths and leads to irreversible signal loss despite refocusing pulses [43,48].

Other cerebral metabolite signals showed a more complex intensity evolution with increasing echo times, i.e., not following a mono-exponential decay (see Figure 2B). These non-mono-exponential decays are well documented in the literature and come from J-coupling effects within the respective spin systems [51,52]. J-coupling between nuclei causes anti-phase and rephasing behavior, as spin interactions cause signal components to evolve anti-phase at TE ≈ 1/2 J^−1^ and rephasing at TE ≈ J^−1^, producing the characteristic oscillatory signal pattern seen in metabolites such as Lac, Tau, and m-Ins. Here, most noticeable, the Lac signal at *δ* = 1.31 ppm (^3^CH_3_) showed an anti-phase pattern between 60 ms and 100 ms and a 180° phase shift at echo times above 100 ms. For Lac, the J-coupling is estimated in the range of 6.9–7.3 Hz, or approximately 1/140 ms [51]. Consequently, the Lac signals are negative in-phase at *TE* = *J*^−1^ ~ 140 ms and anti-phase at *TE* = 1/2 *J*^−1^ ~ 70 ms. This characteristic facilitates distinct lactate identification by applying echo times around 140 ms, but also imposes limitations on echo times due to the complex anti-phase behavior observed within the 60–100 ms echo time range. Other metabolites showing a complex echo time evolution—Tau at *δ* = 3.42 ppm (^1^CH_2_) and *δ* = 3.25 ppm (^2^CH^2^), pCho at *δ* = 3.21 ppm (N(CH_3_)_3_), and Cho at *δ* = 3.19 ppm (N(CH_3_)_3_)—contributed to J-coupling induced signal dephasing. For Tau and m-Ins, the presented signal evolution was consistent with previous observation [53], showing an initial steep signal decline with a local minimum around *TE* = 50–80 ms due to anti-phasing (*TE* ~ 1/2 *J*^−1^), after which the signal recovered between *TE* = 100–140 ms due to rephasing (*TE* ~ *J*^−1^). Estimation of *T*_2_ relaxation times for metabolites with a more complex signal intensity evolution, requires quantum mechanics simulations, as previously reported [53,54]. Overall, our results demonstrate that even at ultra-high fields of 17.6 T and 28.2 T, the *T*_2_ values of the main cerebral metabolites remain sufficiently long to permit reliable identification and quantification. This suggests that the use of specialized sequences designed for very short echo times, such as STEAM, may not always be mandatory at ultra-high field. Instead, moderately longer echo times can be employed to retain adequate metabolite signal while simultaneously suppressing macromolecular contributions, thereby improving spectral clarity and facilitating more accurate metabolite analysis.

### 2.3. Voxel Volume and Averaging Strategies

**Figure 3:**
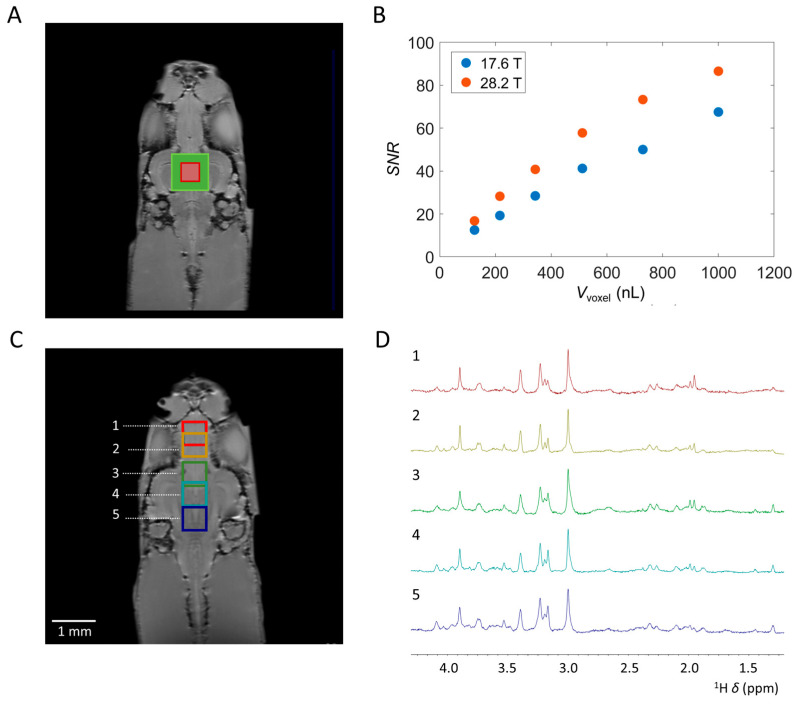
Effect of the voxel volume *V*_voxel_ and voxel positions on localized ^1^H MRS spectra from adult zebrafish brain. (**A**) Anatomical RARE image, coronal slice, of zebrafish brain regions to indicate position of selected PRESS voxels. *V*_voxel_ = 1000 nL (green) and *V*_voxel_ = 125 nL (red) are highlighted. (**B**) Comparison of SNR at 17.6 T and 28.2 T obtained with *ns* = 1024 of the N(CH_3_) tCr signal at *δ* = 3.03 ppm as a function of *V*_voxel_. On average, an SNR increase of 40% was observed at 28.2 T compared to 17.6 T. (**C**) Optimized single voxel localized ^1^H MRS spectra obtained from various locations in the adult zebrafish brain at 28.2 T. Anatomical RARE image, coronal slice, of zebrafish brain regions to indicate position of selected PRESS voxels. (**D**) Highly resolved spectra were obtained from five distinct brain locations. Here, location 1 and 2 are situated in the forebrain, 3, 4, and 5 are situated in the zebrafish mid-brain. Acquisition details unless stated otherwise: *TR* = 2000 ms, *TE* = 15 ms, *ns* = 4096, a voxel size of 0.6 × 0.6 × 0.6 mm^3^ with *V*_voxel_ = 216 nL, 2048 acquisition points, acquisition band-width 11,904 Hz/9.91 ppm, *tBW* = 15 kHz, 4 dummy scans, *v*_1_ = 3 ppm. Water suppression and outer volume suppression were applied as specified in the Materials and Methods Section.

The tiny size of the zebrafish brain, in combination with its shape, limits the utilization of larger voxel sizes. Here, we explored the limits of acquisition of highly resolved single voxel localized MRS spectra in the zebrafish brain in terms of the voxel size and number of averages required for cerebral metabolite identification and quantification. We acquired spectra with *ns* = 1024 in approximately 34 min with *V*_voxel_ ranging between 1000 μL and 125 nL, obtained from the zebrafish midbrain region (Figure 3A) at 17.6 T and 28.2 T. Overall, a noteworthy 40% increase in SNR was observed at 28.2 T compared to 17.6 T (Figure 3B). However, the impact of increased magnetic field strength on elevated *T*_1_ relaxation times was not accounted for [43,55,56]. Hence, it is plausible that a more significant enhancement of the SNR could be achieved by adjusting the repetition time to accommodate the increase in *T*_1_. Given the small size of the zebrafish brain and its predominantly lipid-rich surrounding, elucidated in Supplementary Figure S1, voxels with isotropic dimensions surpassing beyond the brain tissue showed broad and predominant lipid signals. These lipid signals were clearly visible around *δ* = 1.3 ppm and *δ* = 2.0 ppm and overlapped with various cerebral metabolites. In the midbrain, with voxel volumes exceeding *V*_voxel_ = 512 nL (corresponding to a voxel size of 0.8 × 0.8 × 0.8 mm^3^), interference from lipid signals became noticeable. In contrast, for the forebrain and hindbrain, being smaller and more elongated than the midbrain, lipid signal interference became apparent at *V*_voxel_ = 216 nL, corresponding to a voxel size of 0.6 × 0.6 × 0.6 mm^3^. Both at the UHF strength of 17.6 T and 28.2 T, spectra were acquired conducive to the identification and quantification of several cerebral metabolites, even at *V*_voxel_ = 125 nL. To our knowledge, our results represent the use of the smallest *V*_voxel_ demonstrated to date for localized MRS on brain tissue while still allowing for reliable quantification of major brain metabolites. This is significantly smaller than the spatial resolutions typically reported in clinical and preclinical applications. In comparison, for human localized MRS at 3 T, *V*_voxel_ of approximately 4 mL are recommended for reliable quantification of NAA, tCr, and m-Ins [39]. For preclinical applications, *V*_voxel_ in the range of tens of µL are typically utilized [57].

The ability to obtain high-resolution spectra from small PRESS voxels allows for the identification and quantification of cerebral metabolites in various regions in the zebrafish brain. This is elucidated in Figure 3C,D, where at 28.2 T, the cerebral metabolic profiles were obtained from five distinct regions with *V*_voxel_ = 216 nL, corresponding to a voxel size of 0.6 × 0.6 × 0.6 mm^3^, utilizing *ns* = 4096, *tBW* = 15 kHz, and *v*_1_ = 3 ppm. By focusing on specific regions of the zebrafish brain, insight into metabolic processes occurring in that particular area could be obtained, providing valuable information about its function. Furthermore, this particular focus could allow for monitoring metabolic changes in specific brain regions during neurodegenerative processes by utilizing pathological models. Additionally, we explored the number of scans (*ns*) required for identification and quantification of cerebral metabolites in small voxels. At 28.2 T, a voxel volume of 512 nL (0.8 × 0.8 × 0.8 mm^3^) acquired in approximately 2 min enabled the identification and quantification of key cerebral metabolites, including tCr (*δ* = 3.02 and 3.91 ppm), Tau (*δ* = 3.42 and 3.25 ppm), Glx (*δ* = 3.75 ppm), and Lac (*δ* = 1.3 ppm) (see Supplementary Figure S3). Increasing the number of scans significantly improves the spectral quality. This augmentation enables the identification and quantification of additional metabolites, while increasing the SNR in alignment with SNR ~ √*ns*.

### 2.4. Metabolite Identification and Quantification at UHF

**Figure 4:**
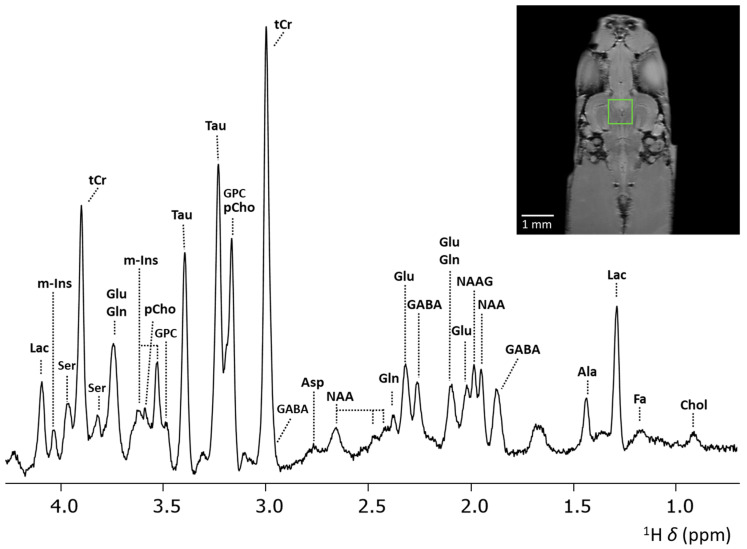
Localized ^1^H MRS spectrum from a voxel (*V*_voxel_ = 512 nL) placed in mid-brain of zebrafish obtained at 28.2 T. Anatomical RARE image, coronal slice, of zebrafish brain regions to indicate position of selected PRESS voxel outlined by the green frame. Acquisition details: *TR* = 2000 ms, *TE* = 15 ms, a voxel size of 0.8 × 0.8 × 0.8 mm^3^ with *Vvoxel* = 512 nL, *ns* = 8192, acquisition points = 2048, acquisition bandwidth 11,904 Hz/9.91 ppm at 28.2 T, 4 dummy scans, *v*_1_ = 3 ppm, *tBW* = 15 kHz. Water suppression and outer volume suppression were applied as specified in the Materials and Methods section. Abbreviations; Lac—Lactate, m-Ins—myo-inositol, tCr—total creatine, Ala—alanine, Glu—glutamate, Gln—glutamine, pCho—phosphocholine, GPC—glycerol-phospho-choline, Tau—taurine, Asp—aspartate, NAAG—N-acetylaspartyl-glutamate, NAA—N-acetylaspartate, GABA—γ-aminobutyric acid, FA—fatty acids, Chol—cholesterol, Ser—Serine.

Figure 4 shows the highly resolved spectrum obtained at 28.2 T from a PRESS voxel with *V*_voxel_ = 512 nL, *tBW* = 15 kHz, and *v*_1_ = 3 ppm. Compared to previous results obtained from in vivo analysis at 400 MHz [29], significantly improved spectra were obtained. This is most evident in the region between *δ* = 1.5–3.0 ppm, where important cerebral metabolite signals are present, e.g., GABA, NAA, NAAG, Glu, and Gln. Previously, the use of lower field strength required a larger PRESS voxel, i.e., *V*_voxel_ = 3375 nL, to acquire ample signal from cerebral metabolites for quantification in the zebrafish brain [29]. Furthermore, spectral resolution is significantly enhanced at 28.2 T compared to lower field strengths. This improvement is clearly demonstrated by the separation of the ^2^CH_3_ signals of NAA and NAAG, which exhibited a chemical shift difference in ∆δ = 0.04 ppm, as expected [58]. NAAG, the most abundant dipeptide neurotransmitter in the brain, is formed in neurons by the coupling of NAA and Glutamate by NAAG synthetases [59] and hydrolyzed by glutamatecarboxypeptidases into NAA and glutamate on the surface of astrocytes [60]. Due to their different function in the central nervous system and their different expression in disease in various brain regions [61,62], quantifying NAA and NAAG separately may provide valuable insights into localized disease mechanisms and improve diagnostic accuracy. However, due to spectral overlap, the ^2^CH_3_ signals of NAA and NAAG are frequently reported as the total NAA (tNAA) [63] signal or simply as NAA [6,7,9,10], given the predominant contribution of NAA in the human brain. Post-processing techniques enable ways to resolve these signals [64], or alternatively, quantification can be achieved through carefully designed sequences specifically for NAA and NAAG detection, such as the MEGA-PRESS sequence by Edden et al. [65]. Previously the separation of NAA and NAAG signals could be achieved using high-resolution localized two-dimensional COSY MRS in mouse brain [66]. As demonstrated here, direct separation of NAA and NAAG signals can be achieved through single voxel localized one-dimensional MRS at UHF strengths.

Besides NAAG, GABA is another important brain metabolite. GABA, the main inhibitory neurotransmitter, plays an important role in memory and learning, motor functions, and neural development [67] and its expression was found to be significantly altered in various neurological disorders [68]. However, due to its relative low concentration and substantial spectral overlap with more abundant metabolites, GABA has been difficult to quantify directly using MRS, often requiring specialized editing techniques to achieve sufficient specificity and accuracy [69]. The direct quantification of GABA has been achieved previously in pre-clinical systems, although some spectral overlap with other metabolites, specifically Glu, was still observed [41]. In this present study, at 28.2 T, we observe a very clear separation of the GABA, Glu, and Gln signals around 2.3 ppm. We achieved almost complete baseline separation of the GABA signal from other metabolites, demonstrating how MRS at ultra-high fields can be utilized not only for its improved SNR, but also for its enhanced spectral resolution. Despite achieving excellent separation of key metabolites at 28.2 T, some signal overlaps are still present. Further refinement may be possible using complementary approaches including localized pure shift NMR, which enhances spectral resolution by removing scalar coupling thereby partly resolving metabolite signals [70] or localized 2D MRS, providing additional information though correlation between nuclei [66].

**Figure 5:**
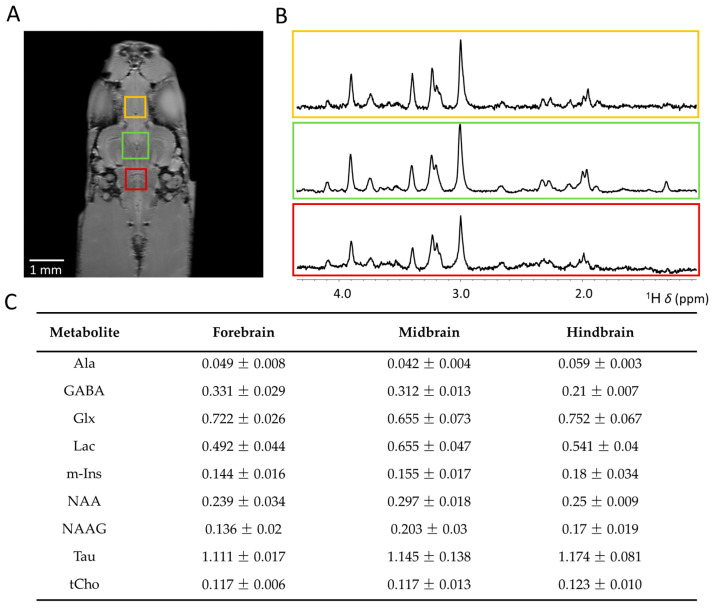
Comparison of cerebral metabolites levels in the forebrain, midbrain, and hindbrain of adult zebrafish at 28.2 T. (**A**) Anatomical RARE image, coronal slice, of zebrafish brain regions to indicate position of selected PRESS voxels in the forebrain, midbrain, and hindbrain. (**B**) Single voxel localized ^1^H MRS spectra obtained from the forebrain, midbrain, and hindbrain. (**C**) Cerebral metabolite levels relative to tCr signal at *δ* = 3.03 ppm, *n* = 3. Yellow labels correspond to the forebrain, green to the midbrain, and red to the hindbrain. Glx = (Glu + Gln), tCho = (GPC + pCho).

In Figure 5, we present quantification analysis of the cerebral metabolic profile obtained from different regions in the zebrafish brain (see Figure 5A), acquired at 28.2 T. For the midbrain, spectra were acquired in approximately 15 min using a voxel volume of 512 nL, while for the forebrain and hindbrain, acquisition took around 30 min with a voxel volume of 216 nL. These relatively short measurement times show the potential feasibility of applying our approach in vivo, while still allowing for the quantification of most major brain metabolites. The importance of moving towards in vivo applications is highlighted by the relative low levels of NAA found, compared to values reported earlier in vivo and in brain extracts [29]. These low levels of NAA can be attributed to degradation processes in the first few hours postmortem, as previously shown in rodent brain tissue [71]. For systems where in vivo measurements are not feasible, an alternative approach is to preserve of the postmortem metabolic profile using in situ microwave fixation, as previously demonstrated in the rodent brain [72]. Nonetheless, highly resolved spectra were obtained from each region (see Figure 5B). Quantification of metabolites is shown in Figure 5C. We observed minimal variation in metabolite levels across the studied brain regions. However, significantly lower levels of GABA were found in the hindbrain compared to the forebrain (*p* < 0.005) and midbrain (*p* < 0.05). Throughout the zebrafish brain, GABAergic neurons are not uniformly distributed. The hindbrain voxel, situated just posterior to the cerebellar corpus, overlaps with several relatively large white matter structures in the zebrafish brain, including the lateral longitudinal fascicle (LLF), ventral rhombencephalic commissure (Cven), and the medial longitudinal fascicle (MLF) [73,74], likely contributing to the relative low GABA levels, as white matter contains few GABAergic neurons and mainly functions to transmit axonal signals rather than mediate local inhibitory activity [75]. On the contrary, the voxel placed in the forebrain and midbrain overlap with regions including the optic tectum, the subpallium, and the corpus cerebelli, exhibiting higher densities of GABAergic neurons [76,77], contributing to inhibitory regulation essential for the refined sensory input, coordinated movement, and excitatory network stability regulated in these structures [78].

## 3. Materials and Methods

### 3.1. Zebrafish Husbandry

The husbandry of the zebrafish was performed according to the regulations set forth by the local animal welfare committee of Leiden University (License numbers AVD1060020171767 and AVD10600202216175), in accordance with the international standards outlined by the EU Animal Protection Directive 2010/63/EU. All procedures followed standard protocols as previously described (www.zfin.org) [79]. In this study, three-month-old male zebrafish were utilized. To minimize any metabolite degradation, zebrafish were euthanized just before the MRI examination, which was induced by immobilization through submersion in ice water at a temperature between 0 and 4 °C for 15 min until the halt of opercular movement. Following euthanization, specimens were shortly kept on ice until MRI and MRS measurements were conducted.

### 3.2. Magnetic Resonance Imaging and Spectroscopy

MRI and MRS experiments were conducted using Bruker (Bruker Biospin GmbH, Mannheim, Germany) vertical bore systems operating at 17.6 T (750 MHz) or 28.2 T (1200 MHz), configured with a Micro5 or Micro5 iProbe gradient system, respectively. Both systems were water-cooled, provided G_max_ = 3 T/m, used 5 mm inner diameter birdcage resonators, and GREAT60 gradient power supplies. Measurements were conducted actively maintaining room-temperature (20 °C). Data acquisition and processing on both systems was performed with Linux workstations, running Bruker imaging software ParaVision 360 v3.3 (Bruker Biospin GmbH, Mannheim, Germany). During analysis, zebrafish were embedded in perfluoropolyether (Fomblin Y, Solvay Solexis S.P.A., Bollate, Italy).

*T*_2_-weighted spin echo images were acquired by a rapid acquisition with relaxation enhancement (RARE) pulse sequence to select the position for single voxel localized MRS. RARE measurements were performed with an echo time *TE* = 5.6 ms, a repetition time *TR* = 3000 ms, using 4 refocusing pulses per excitation, and *ns* = 16 averages. The field of view (FOV) was 8 mm × 8 mm, at a matrix size of 256 voxels × 256 voxels, obtaining a resolution of 31 μm × 31 μm, with a slice thickness of 200 μm.

For single voxel localized ^1^H MRS, we utilized a PRESS pulse sequence [36]. The PRESS sequence incorporated three transmitter slice-selective RF pulses (90°-180°-180°), applied simultaneous with *x*, *y*, and *z* gradients to target the specified voxel. Prior to each localized MRS measurement, magnetic field homogeneity within each voxel was achieved by local second-order shimming prior to data acquisition. After parameter optimization, standard PRESS measurements were performed with *TE* = 15 ms, *TR* = 2000 ms, *ns* = 512. The PRESS sequence used 2048 complex points and an acquisition bandwidth of 10 ppm. At 17.6 T and 28.2 T, the transmitter RF pulse bandwidth was *tBW* = 10 kHz or 15 kHz, respectively. The excitation frequency was *v*_1_ = 3 ppm, coinciding with the N(CH_3_) total creatine (tCr) signal. For the estimation of regional cerebral metabolite levels, three voxels were selected in the zebrafish brain: (1) in the forebrain, covering part of the pallium; (2) in the midbrain, covering part of the mesencephalon, cerebellum, and diencephalon; (3) in the hindbrain, covering part of the cerebellum and rhombencephalon. In the midbrain, a PRESS voxel of 0.8 × 0.8 × 0.8 mm^3^, with a voxel volume of *V*_voxel_ = 512 nL was selected. In the forebrain and hindbrain, a PRESS voxel of 0.6 × 0.6 × 0.6 mm^3^, with *V*_voxel_ = 216 nL was selected. For suppression of the water signal, a variable pulse power and optimized relaxation delays (VAPOR) sequence [80] was applied prior to the PRESS pulses. Outer volume suppression (OVS) was integrated in an interleaved manner with the VAPOR sequence. OVS utilized hyperbolic secant pulses and consisted of six suppression slices, each repeated three times and aligned parallel to the PRESS voxel, reducing the PRESS gradient spoilers. Each suppression slice had a thickness of 5 mm with an inter-slice gap of 0.1 mm, and employed spoiler gradients with a duration of 1 ms and a strength of 40 % × 40 % × 40% along the three gradient axes. In Paravision 360 v3.3, automatic eddy current compensation was activated, using the unsuppressed water reference scan to correct phase distortions, effectively reducing line shape distortions caused by eddy currents.

### 3.3. Data Processing

Identification of cerebral metabolites was performed by comparison of obtained ^1^H spectra to reference spectra of brain metabolites [58,81,82] and on our previously obtained NMR spectra of zebrafish brain extracts [29]. Quantification of metabolites was executed using Chenomx NMR suite 8.2 (Chenomx Inc., Edmonton, AB, Canada), using the following signals per metabolite: Alanine (Ala) ^3^CH_3_ at *δ* = 1.47 ppm; γ-aminobutyric acid (GABA) ^3^CH_2_ at *δ* = 1.89 ppm; Glutamate (Glu) ^4^CH_2_ at *δ* = 2.45 ppm; Glutamine (Gln) ^2^CH at *δ* = 3.75 ppm; Lactate (Lac) ^2^CH at *δ* = 4.09 ppm; *myo*-Inositol (m-Ins) at *δ* = 3.61 ppm; N-acetylaspartate (NAA) ^2^CH_3_ at *δ* = 2.01 ppm; N-acetylaspartate-glutamate (NAAG) ^2^CH_3_ at *δ* = 2.01 ppm; taurine (Tau) ^2^CH_2_ at *δ* = 3.25 ppm; total choline (tCho = GPC + pCho) N(CH_3_)_3_ at *δ* = 3.20 ppm. Available reference libraries provided by Chenomx were utilized. Metabolite concentrations were estimated relative to tCr [83]. Statistical analysis for NMR quantification involved one-way analysis of variance (ANOVA) by OriginPro v.8 (Originlab, Northhampton, MA, USA). Multiple comparisons were conducted using *t*-test, and significance was established with *p* < 0.05. Unless specified otherwise, SNR was estimated based on the tCr signal at a chemical shift *δ* = 3.03 ppm, utilizing built-in TopSpin functionalities with

*SNR* = *I*_tCr_/(2∙*N*). (1)

Here, ItCr is the maximum signal intensity of the tCr signal at *δ* = 3.03 ppm, and *N* is the noise factor as fully described in the TopSpin manual (Processing commands and parameters user manual, version 007, Bruker Corporation, Mannheim, Germany). In this present study, presented *CSD* was based on the estimation for a single spatially selective pulse (CSD_pulse_), given by

*CSD*_pulse_ (%) = |*v*_1_ − *v*_x_|/*tBW*, (2)

where *v*_x_ is the Larmor frequency of the metabolite of interest (Hz). Additionally, since the PRESS sequence utilizes three spatially selective pulses, the total CSD for the whole voxel can be estimated by

*CSD*_voxel_ (%) = (|*v*_1_ − *v*_x_|/*tBW*)^3^, (3)

## 4. Conclusions and Future Outlook

This study reported the first successful application of localized MRS at 28.2 T, the highest commercially available magnetic field strength to date. We achieved localized MRS in extremely small brain regions, with total scan times between 15 and 30 min, making the technique suitable for in vivo use. Our protocol enabled accurate quantification of key brain metabolites and produced high-quality spectra from voxel volumes as small as 125 nL. This advancement in spatial resolution of localized MRS at 28.2 T, opens up new opportunities for exploring regional neurochemical profiles in the zebrafish brain using various zebrafish models of human diseases.

In future, our optimized methods, with relatively short measurement times of up to 30 min, are well-suited for implementation in in vivo studies. In vivo measurements will enable the utilization of various zebrafish models of human pathological diseases for longitudinal studies by allowing for continuous and non-invasive monitoring of metabolic changes over time. This approach will facilitate the study of disease progression and the long-term effects of therapeutic interventions within the same subjects, providing a more comprehensive understanding of the underlying mechanisms. Although the current narrow-bore 28.2 T imaging system presents challenges for in vivo measurements on zebrafish due to space constraints, in vivo studies can be implemented, e.g., using human organoid-based models [84], for detailed metabolic investigations that remain highly relevant to human pathological conditions and can effectively complement future in vivo applications. Additionally, future work at 28.2 T could explore alternative methods for localized spectroscopy that are less susceptible to CSD effects, including adiabatic selective excitation refocusing (LASER), semi-LASER, stimulated echo acquisition mode (STEAM), and image-selected in vivo spectroscopy (ISIS), each of which presents complementary trade-offs. More advanced techniques, such as localized two-dimensional COSY and diffusion-weighted MRS, could also be investigated to further enhance metabolic specificity and microstructural sensitivity. Furthermore, in the present work, metabolite quantification was performed using Chenomx NMR Suite. While this approach proved suitable for our pilot study, it is susceptible to user variability and lacks the degree of automation offered by state-of-the-art tools such as LCModel [85]. Future studies should therefore aim to implement LCModel or comparable software, utilizing simulated model spectra for each metabolite and echo time rather than relying on individual resonance fitting in order to provide a more robust and quantitative analysis of metabolite concentrations.

## Data Availability

The data presented in this study are available on request from the author (A.A.).

## Supplementary Materials

The following supporting information can be downloaded at: https://www.mdpi.com/article/10.3390/molecules30214320/s1, Figure S1. Chemical-shift-selective imaging (CSSI) for the visualization of lipid distribution in the zebrafish head at 28.2 T. Figure S2. Full comparison of echo times in single voxel localized ^1^H MRS spectrum from adult zebrafish midbrain at 17.6 T and 28.2 T, in the range between 15 ms and 144 ms. Figure S3. Impact of *ns* in localized ^1^H MRS from adult zebrafish mid-brain at 28.2 T.

## Abbreviations

The following abbreviations and symbols are used in this manuscript:

B_0_	Magnetic field strength
Cr	Creatine
*G*	Magnetic field gradient strength
GABA	γ-aminobutyric acid
Gln	Glutamine
Glu	Glutamate
HR-MAS	High-resolution magic angle spinning
m-Ins	Myo-inositol
MRI	Magnetic resonance imaging
MRS	Magnetic resonance spectroscopy
NAA	N-acetylaspartate
NAAG	N-acetylaspartyl-glutamate
*ns*	Number of scans
PRESS	Point resolved spectroscopy
RARE	Rapid acquisition with relaxation enhancement
SNR	Signal-to-noise ratio
*CSD*	Chemical shift displacement
*T*_2_	Transverse relaxation time
Tau	Taurine
*tBW*	Transmitter RF pulse bandwidth
*TE*	Echo time
*TR*	Repetition time
UHF	Ultra-high field
*v*_1_	Excitation frequency
VAPOR	Variable pulse power and optimized relaxation delays
*V*_voxel_	Voxel volume
*v*_x_	Larmor frequency compound x
*δ*	Chemical shift
