# Supplementary material for: Non-Invasive Regional Neurochemical Profiling of Zebrafish Brain Using Localized Magnetic Resonance Spectroscopy at 28.2 T
Source: Molecules. 2025 Nov 6;30(21):4320. doi: 10.3390/molecules30214320 (PMC12610310; doi:10.3390/molecules30214320)
Supplement: Supplementary file 1 [file molecules-30-04320-s001.zip › molecules-3865091-supplementary.pdf]

Non-Invasive Regional Neurochemical Profiling of Zebrafish Brain Using Localized Magnetic Resonance Spectroscopy at 28.2 T

Rico Singer [1], Wanbin Hu [2], Li Liu [2], Huub J. M. de Groot [1], Herman P. Spaink [2] and A. Alia [1,3,*]

[1] Leiden Institute of Chemistry, Leiden University, Einsteinweg 55, 2301 RA Leiden, The Netherlands; r.singer@lic.leidenuniv.nl (R.S.); groot_h@lic.leidenuniv.nl (H.J.M.d.G.)
[2] Institute of Biology, Leiden University, Einsteinweg 55, 2301 RA Leiden, The Netherlands; w.hu@biology.leidenuniv.nl (W.H.); l.liu@biology.leidenuniv.nl (L.L.); h.p.spaink@biology.leidenuniv.nl (H.P.S.)
[3] Institute of Medical Physics and Biophysics, Leipzig University, Härtelstr. 16-18, D-04107 Leipzig, Germany
* Correspondence: a.alia@chem.leidenuniv.nl or alia.aliamatysik@medizin.uni-leipzig.de

Supporting Information Available

Figure S1. Chemical shift selective imaging (cssi) for the visualization of lipid distribution in the zebrafish head at 28.2 T.

Figure S2. Full comparison of echo times in single voxel localized 1H MRS spectrum from adult zebrafish midbrain at 17.6 T and 28.2 T, in the range between 15 ms and 144 ms.

Figure S3. Impact of *ns* in localized ^{1}H MRS from adult zebrafish mid-brain at 28.2 T.

 https://doi.org/10.3390/molecules30214320

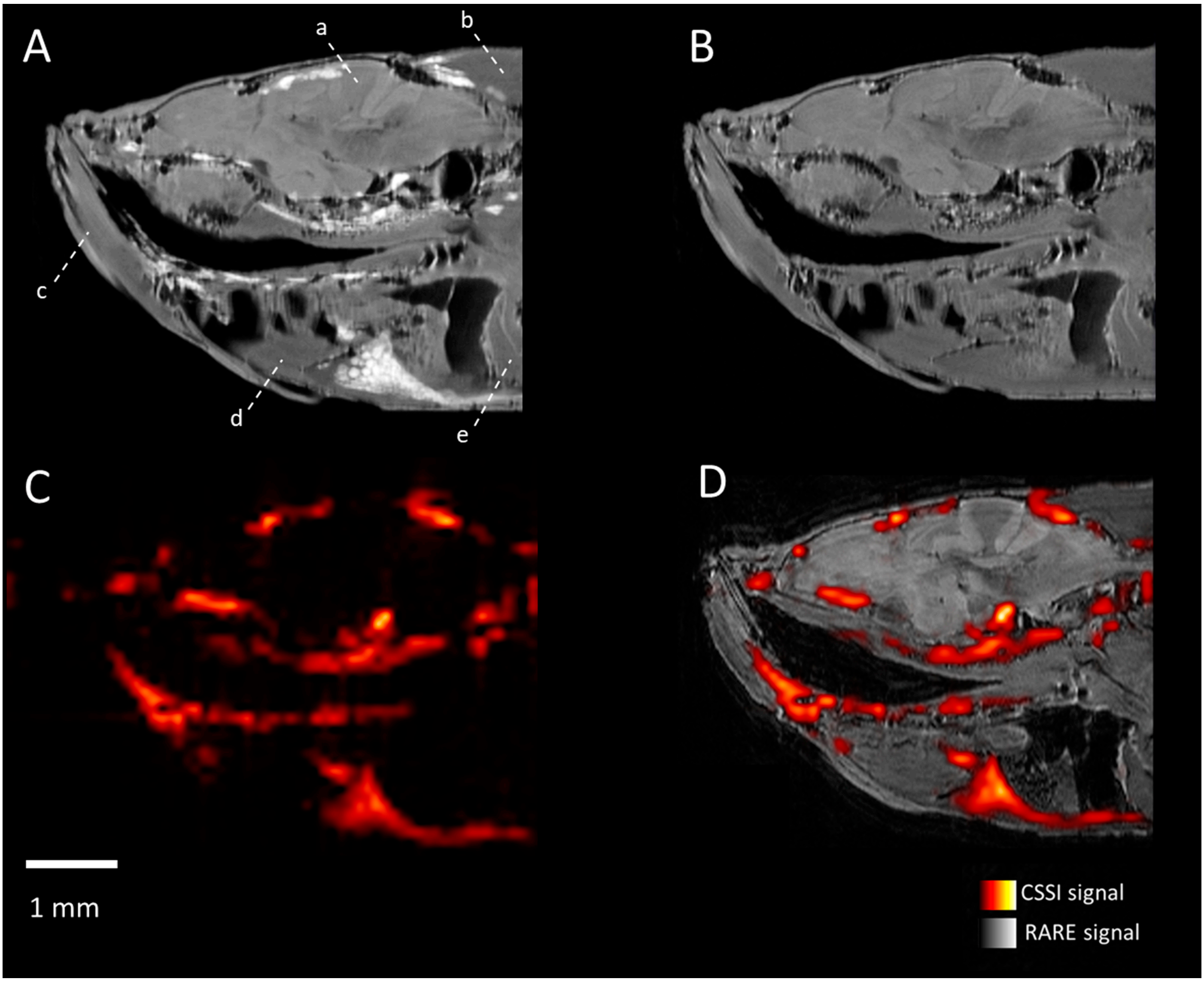

Figure S1. **Chemical shift selective imaging (cssi) for the visualization of lipid distribution in the zebrafish head at 28.2 T.** For these measurements, wild-type three-month-old adult-zebrafish, fixed in 4% buffered paraformaldehyde were utilized as described previously. **[A]** Sagittal 2D RARE image without fat suppression. Chemical shift artifact causes displacement of lipids in the frequency-encode direction, which increases with $\mathbf{B}_0$. **[B]** Sagittal 2D RARE image with fat suppression. **[C]** Lipid signal obtained by 3D cssi. **[D]** 3D cssi signal (red color scale) overlayed on 3D RARE image (grey color scale), showing the distribution of lipids in the zebrafish head. Data acquisition and processing were performed using Paravision 360 v3.3. Acquisition details: 2D RARE images: *TR* = 3000 ms, *TE* = 5.6 ms, *ns* = 64, resolution 23 µm × 23 µm × 100 µm, RARE factor 4. 3D RARE image: *TR* = 1800 ms, *TE* = 6.1 ms, *ns* = 4, resolution 23 µm × 23 µm × 47 µm, RARE factor 16. 3D cssi image: *TR* = 2000 ms, *TE* = 4.5 ms, *ns* = 1, resolution 94 µm × 94 µm × 188 µm, shift selective excitation frequency 1200.7 MHz (1.24 ppm), excitation pulse bandwidth 2000 Hz (1.6 ppm). (a) brain; (b) muscle; (c) dentary; (d) sternohyoid; (e) liver

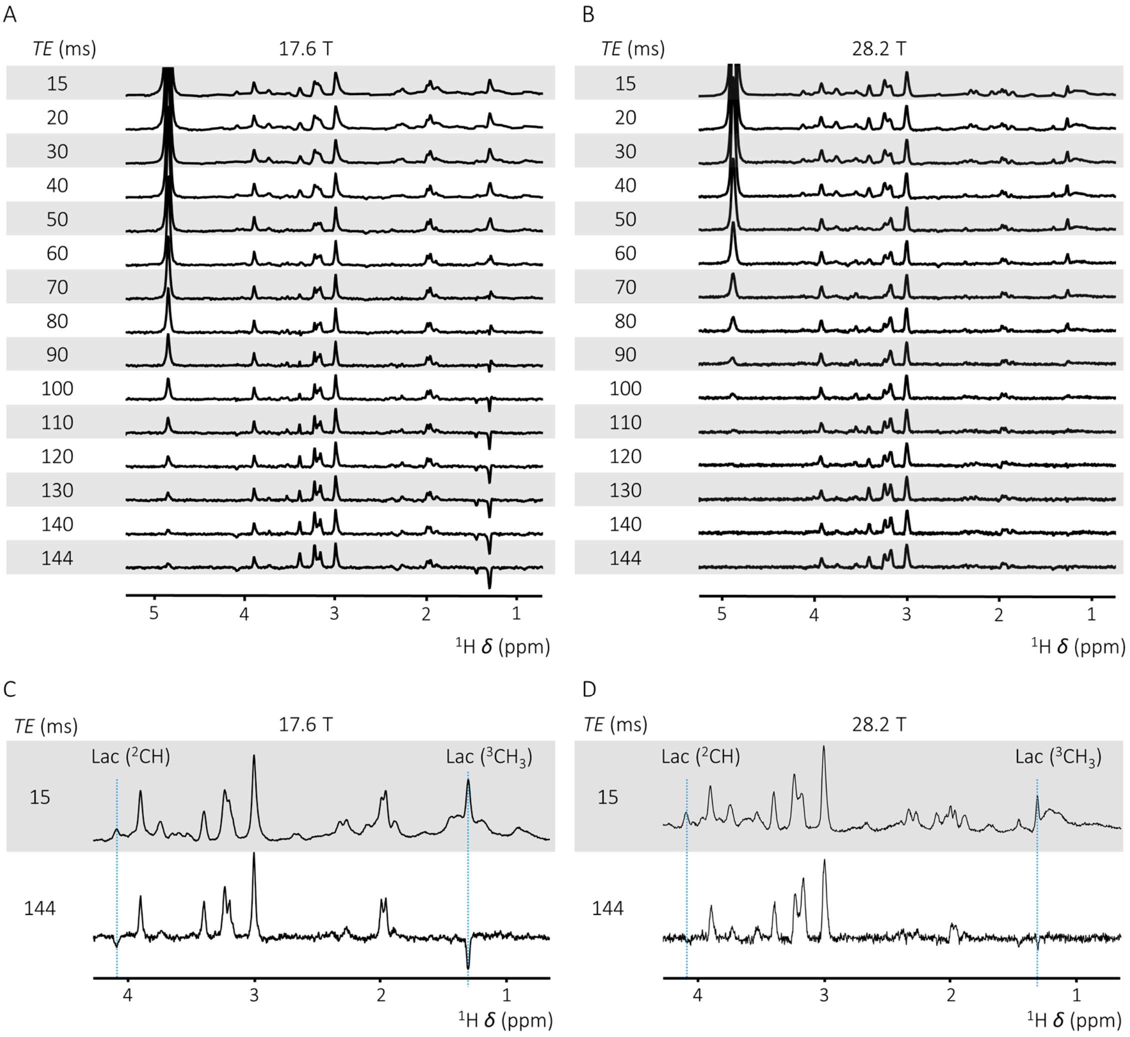

E

Metabolite	δ (ppm)	T_2 at 28.2 T (ms)*	T_2 at 17.6 T (ms)*
tCr	3.03	75.5	112.4
Glx	3.75	24.8	33.4
NAAG	2.04	99.8	149.4
NAA	2.01	47.7	51.8
GABA	1.89	25.3	25.2

Figure S2. Full comparison of echo times in single voxel localized ^{1}H MRS spectrum from adult zebrafish midbrain at 17.6 T and 28.2 T, in the range between 15 ms and 144 ms. [A – B] Single voxel localized MRS spectra acquired for echo times at 17.6 T and 28.2 T, respectively. **[C - D]** Comparison of spectra obtained at a short echo time of 15 ms and a long echo time of 144 ms at 17.6 T and 28.2 T. Particular attention is directed towards the lactate 180° phase shift at 144 ms, contributed to J-coupling induced signal dephasing. This effect is prominently evident at 17.6 T. However, at 28.2 T, due to escalated chemical shift displacement effects, the lactate signal acquired at 144 ms became indistinguishable from spectral noise, highlighting the necessity for compensation strategies to address these effects. **[E]** T_2 relaxation times estimated for brain metabolites by mono-exponential line fitting at 28.2 T and 17.6 T. T_2 relaxation times of individual metabolites were estimated using PRESS signal intensities acquired at various echo times. T_2 was estimated using a non-linear least squares algorithm to fit the mono-exponential function described by $I_{TE} = A + I_0 \cdot \exp(-TE / T_2)$. Here, I_{TE} represents the signal intensity at TE, A is the absolute bias, and I_0 is the signal intensity at TE_0. *Measurements were performed with $n = 1$ as a proof-of-principle.

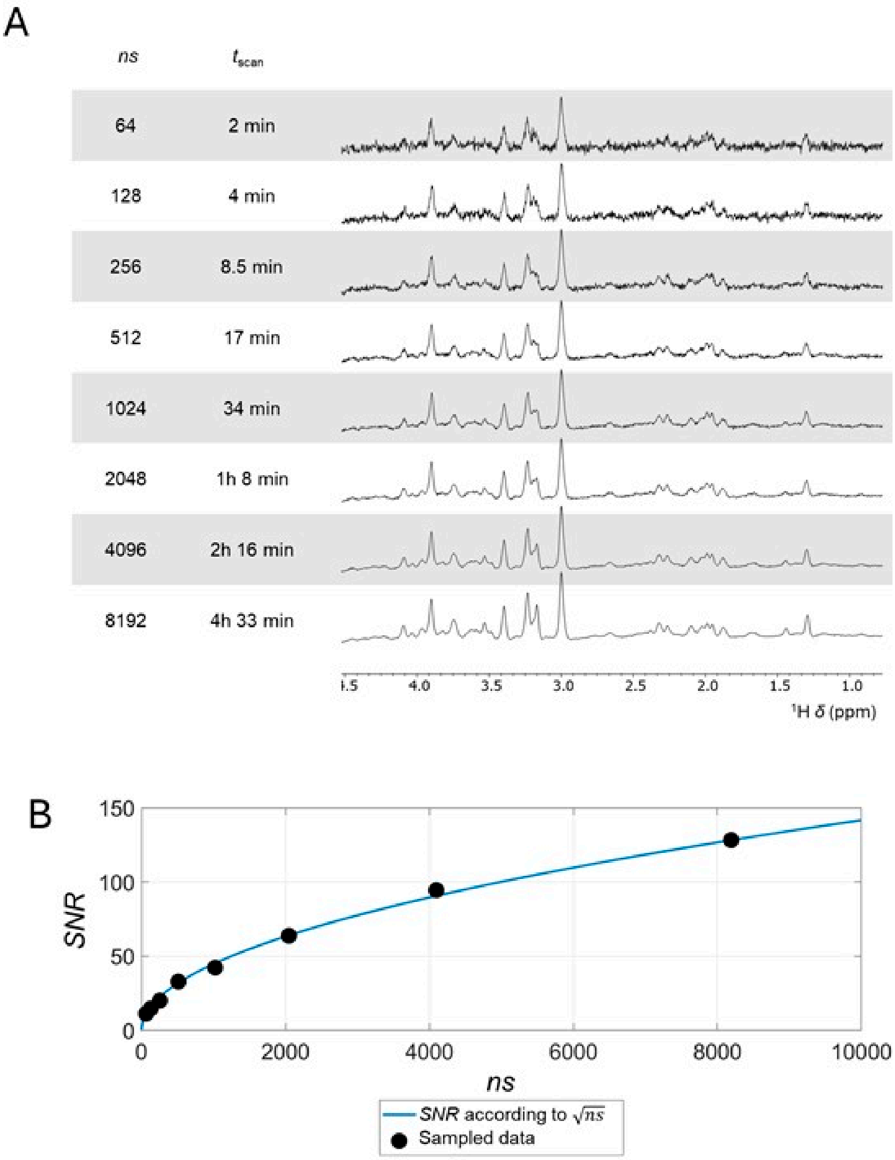

Figure S3. Impact of *ns* in localized ^{1}H MRS from adult zebrafish mid-brain at 28.2 T. [A] Single voxel localized MRS spectra acquired from the mid-brain of an adult zebrafish, acquired with increasing *ns* [B] SNR of the tCr signal at δ = 3.03 ppm as a function of *ns*. Acquisition details: *TR* = 2000 ms, *TE* = 15 ms, a voxel size of 0.8 × 0.8 × 0.8 mm^3 with V_{voxel} = 512 nL, 2048 acquisition points, acquisition bandwidth 11904 Hz / 9.91 ppm, *tBW* = 15 kHz, 4 dummy scans, υ_1 = 3 ppm. Water suppression and outer volume suppression were applied as specified in the method section.
